# Clinical Study of Nasopharyngeal Carcinoma Treated by Helical Tomotherapy in China: 5-Year Outcomes

**DOI:** 10.1155/2014/980767

**Published:** 2014-07-09

**Authors:** Lei Du, Xin-Xin Zhang, Lin Ma, Lin-Chun Feng, Fang Li, Gui-Xia Zhou, Bao-Lin Qu, Shou-Ping Xu, Chuan-Bin Xie, Jack Yang

**Affiliations:** ^1^Department of Radiation Oncology, Chinese PLA General Hospital, 28 Fuxing Road, Beijing 100853, China; ^2^Department of Radiation Oncology, Hainan Branch of Chinese PLA General Hospital, Haitang Bay, Sanya 572000, China; ^3^Department of Otorhinolaryngology, Chinese PLA General Hospital, 28 Fuxing Road, Beijing 100853, China; ^4^Department of Medical Oncology, Chinese PLA General Hospital, 28 Fuxing Road, Beijing 100853, China; ^5^Department of Radiation Oncology, Monmouth Medical Center, 300 2nd Avenue, Long Branch, NJ 07740, USA

## Abstract

*Background.* To evaluate the outcomes of nasopharyngeal carcinoma (NPC) patients treated with helical tomotherapy (HT). *Methods.* Between September 2007 and August 2012, 190 newly diagnosed NPC patients were treated with HT. Thirty-one patients were treated with radiation therapy as single modality, 129 with additional cisplatin-based chemotherapy with or without anti-EGFR monoclonal antibody therapy, and 30 with concurrent anti-EGFR monoclonal antibody therapy. *Results.* Acute radiation related side effects were mainly grade 1 or 2. Grade 3 and greater toxicities were rarely noted. The median followup was 32 (3–38) months. The local relapse-free survival (LRFS), nodal relapse-free survival (NRFS), distant metastasis-free survival (DMFS), and overall survival (OS) were 96.1%, 98.2%, 92.0%, and 86.3%, respectively, at 3 years. Cox multivariate regression analysis showed that age and T stage were independent predictors for 3-year OS. *Conclusions.* Helical tomotherapy for NPC patients achieved excellent 3-year locoregional control, distant metastasis-free survival, and overall survival, with relatively minor acute and late toxicities. Age and T stage were the main prognosis factors.

## 1. Background

Nasopharyngeal carcinoma (NPC) is one of the most common head and neck cancers in China, with an incidence ranging from 25 to 30 out of 100,000 per year [1]. Due to the anatomical and biological specificity of NPC, radiation therapy or chemoradiotherapy has been recognized as an effective radical treatment. Unlike two-dimensional conventional radiation therapy (2DCRT) and three-dimensional conformal radiation therapy (3DCRT), intensity modulated radiation therapy (IMRT) can deliver a highly conformed dose to targets while effectively sparing critical normal organs and has the potential to improve local control rate and reduce radiation-related toxicities [2–11]. The possibility of dose-painting, optimized dose distributions, and improved treatment outcome have shifted the treatment of choice for NPC toward IMRT.

Helical tomotherapy (HT) is a unique IMRT modality that combines elements of diagnostic radiology and radiation therapy in a single unit. In addition to the ability to deliver a highly conformal dose distribution, HT is equipped with xenon detectors designed to obtain megavoltage computed tomography (MVCT) images utilized for pretreatment setup verification [12]. This system has many dosimetric advantages, but to date few clinical observations of large number of NPC patients treated with HT-based IMRT have been reported [13–15]. Since our center installed the first HT unit in China in September 2007, more than 1800 cases have been treated. Among these patients 40% were head and neck cancers. After the report of our short-term observation of 73 NPC patients [15], we present here the clinical observation of 190 NPC patients treated with the only HT unit in China during 5 years.

## 2. Materials and Methods

### 2.1. Patient's Characteristics

Over five years, from September 2007 to August 2012, there were 190 histologically proven nonmetastatic NPC patients treated with HT at our center. All patients had nasopharyngeal and skull base computed tomography (CT) or magnetic resonance imaging (MRI), endoscopic evaluation, complete blood counts, hepatic and renal function tests, neck and abdomen ultrasound, and bone scanning. Positron emission tomography (PET) was optional. Clinical stage was established according to the UICC 2002 staging system (Table 1).  Table 2 summarizes patients' characteristics. Informed consent was obtained from all patients before receiving treatment, and this study was approved by the ethics committee of the Chinese PLA General Hospital.

### 2.2. Helical Tomotherapy

Plain and enhanced CT images with 3 mm slice thickness were taken for treatment planning, then transmitted to the Pinnacle^3^ 8.0 workstation, and fused. Enhanced CT, MRI, or PET images were used as a guide for target contours. The gross target volume of the primary tumor (GTVnx) and metastatic lymph nodes (GTVnd) were, respectively, defined as the visible tumor and involved nodes larger than 1 cm in diameter or with necrotic centers on CT or MRI images. The pGTVnx was obtained by expanding the corresponding GTVnx with a margin of 3–5 mm limited by the brainstem, spinal cord, optic chiasma, and optic nerve. The pGTVnd was the GTVnd with an expansion of 3 mm. The clinical target volume 1 (CTV1) covered nasopharynx, high-risk local structures (i.e., skull base, clivus, parapharyngeal space, retropharyngeal lymph nodes, sphenoid sinus, sphenomaxillary fossa, posterior part of the nasal cavity and maxillary sinus, and oropharynx), and positive lymph nodes and nodes at levels IB (when nodes at level II were involved) and II and the superior part of VA. CTV2 included lymph nodes at levels III, IV, and VB and the inferior part of VA as a prophylactic irradiated volume. Each CTV was automatically expanded to generate the corresponding planning target volume (PTV) with an isotropic 3 mm margin while assuring the edge of the distribution was at least 2 mm from skin. The organs at risk (OAR), including pituitary gland, brainstem, eyeballs, lens, optic nerves, spinal cord, temporomandibular joints, inner ears, parotid glands, oral cavity, and larynx-esophagus-trachea, were also delineated. In areas where the target volume was adjacent to critical normal structures, the margin was accordingly reduced. The CT images with the contoured structures were transferred to the HT (Hi Art TomoTherapy 2.2.4.1) workstation.

The planning dose at D95 was prescribed to pGTVnx and pGTVnd at 70–74 Gy, PTV1 at 60–62.7 Gy, and PTV2 at 52–56 Gy in 33 fractions. No more than 5% of PTV volume received more than 110% of the prescribed dose. Based on RTOG H-0022 protocol [16] and our own experiences, the following dose-volume constraints for OARs were utilized: (1) brainstem *D*_max⁡_ < 54 Gy; (2) lens *D*_max⁡_ < 5 Gy; (3) optic nerve *D*_max⁡_ < 54 Gy; (4) spinal cord *D*_max⁡_ < 45 Gy; (5) temporomandibular joint *D*_max⁡_ < 60 Gy; (6) inner ear *D*_max⁡_ < 60 Gy; (7) parotid gland V30 < 50% or *D*_mean_ < 28 Gy; (8) oral cavity V40 < 30%; and (9) larynx-esophagus-trachea V40 < 30%. HT plans were developed based on a field width of 2.5 cm, a pitch of 0.30–0.38, and a modulation factor of 2.0–3.0.

During HT therapy, patients underwent MVCT imaging at least once every week to verify patient setup. The imaging frequency was determined by the magnitude of setup errors from initial daily scans. Since March 2009 MVCT image-guidance was performed before each fraction of HT therapy. Automatic and manual registration of the MVCT images with the planning CT images was based on bony and tissue anatomy. Radiation therapy was delivered once daily, 5 days per week.

### 2.3. Chemotherapy and Anti-EGFR Monoclonal Antibody Treatment (Table 3)

Thirty-one patients were treated with HT-based radiation therapy as the sole modality, 129 cases received additional cisplatin-based chemotherapy with or without concurrent anti-EGFR monoclonal antibody (Mab) treatment, and 30 cases were treated with concurrent anti-EGFR Mab therapy. Neoadjuvant chemotherapy consisted of 1-2 cycles of DP (docetaxel 75 mg/m^2^, d1, cisplatin 80 mg/m^2^, d1 and every 3 weeks) or a single DDP regimen. According to clinical stages, tolerance and economic status, concurrent chemotherapy, and/or anti-EGFR Mab treatment were performed in one of four patterns: (1) cisplatin 80 mg/m^2^, d1, every 3 weeks; (2) docetaxel 60 mg/m^2^, d1, and cisplatin 60 mg/m^2^, every 3 weeks; (3) cetuximab 250 mg/m^2^ or nimotuzumab 200 mg, d1, every week; (4) cetuximab 250 mg/m^2^ or nimotuzumab 200 mg, d1, every week and cisplatin 80 mg/m^2^, d1, every 3 weeks. Adjuvant chemotherapy consisted of 4~6 cycles of DP regimen. No more than 6 cycles of chemotherapy (including neoadjuvant, concurrent, and adjuvant patterns) were given for each patient.

### 2.4. Follow-Up

Acute side effects were investigated weekly and peak toxicities were recorded. Acute and late side effects were defined and graded according to the established RTOG/EORTC criteria [17]. The preliminary response was evaluated one month after the end of radiation therapy. Patients' follow-up examinations were conducted every 3 months for the first year and subsequently every 6 months to evaluate therapeutic effects. The median follow-up was 32 months, ranging from 6 to 58 months from the beginning of radiation therapy, with a follow-up rate of 100%. Local relapse-free survival (LRFS), nodal relapse-free survival (NRFS), distant metastases-free survival (DMFS), and overall survival (OS) were analyzed by the Kaplan-Meier method. Different prognostic factors were analyzed by log-rank test and the Cox proportional hazards model was used for multivariate analysis. *P* < 0.05 was considered significant. The analyses were performed with the SPSS software package (Version 19.0, SPSS Inc., Chicago, IL).

## 3. Results

### 3.1. Dosimetric Analysis

The average length of treatment in the superior-inferior plane was 22.9 cm (17.0–28.7 cm) and average beam-on time was 456.5 s (358.0–696.1 s). The mean dose to pGTVnx, pGTVnd, PTV1, and PTV2 was 72.5 Gy, 72.3 Gy, 64.7 Gy, and 56.9 Gy, respectively.

The delivered doses to OARs generally met the established constraints. The mean dose to left and right parotid gland was 31.0 Gy and 30.8 Gy, respectively. The mean doses to bilateral temporomandibular joints, oral cavity, and larynx-esophagus-trachea were less than 40 Gy. The maximum dose to brainstem and spinal cord was 54.6 Gy and 41.2 Gy, respectively.

### 3.2. Acute and Late Side Effects

Radiation therapy, which was complete in all 190 patients, was interrupted for 1 week in four cases because of grade 3 acute skin toxicity; among these patients, three were in the concurrent chemoradiotherapy group (DTX-CDDP) and one in the radiation therapy single modality regiment. Concurrent chemotherapy (DTX-CDDP) was stopped after 1 cycle in a single patient due to a grade 4 leucopenia. Drug doses were reduced in two female patients because of a grade 3 leucopenia and a grade 3 pharynx-esophageal toxicity after 1 cycle of concurrent chemotherapy (DTX-DDP). Acute radiation related side effects were mainly of grade 1 or 2 in skin, oral mucosa, salivary glands, and pharynx-esophagus. Grade 3 toxicities were noted in six cases for skin, eight for mucosa, and one for pharynx-esophagus. By the completion of radiation therapy, patients lost 11.5% of their pretreatment weight on the average, ranging from −0.2% to 25.0%. Distributions of acute side effects in different treatment groups are shown in the Table 4.

Fifty-one percent of cases underwent different degrees of cutaneous dropsy in the maxillofacial region and 54.2% of them recovered completely on the average of 5.8 months after radiation therapy. Thirty-seven cases underwent otitis media, of which ten needed surgical treatment. Fifty-seven patients (*D*_mean_ of inner ear was 48.1 Gy) suffered from grade 1 to 2 hearing loss. Twenty patients had a difficulty in opening mouth (*D*_mean_ of temporomandibular joints was 40.3 Gy). Two patients complained of tooth looseness. Two patients underwent radioactive pulpitis which was treated by surgical operation. Six patients reported a diminished sense of taste. Xerostomia was slowly restored with passing of time. No grade 2 or more xerostomia was noted one year after radiation therapy.

### 3.3. Patterns of Failure (Table 5)

One month after radiation therapy, evaluation of primary lesions showed 95 complete responses (CR), 88 partial responses (PR), and 7 stable diseases (SD); evaluation for involved lymph nodes in 149 patients showed 84 CR, 61 PR, and 4 SD, with a remission rate of 96.3% and 97.3% for primary lesions and involved lymph nodes, respectively. No progressive disease (PD) was noted.

Eight patients (70 Gy/33 F to pGTVnx) had a pathologically confirmed recurrence in the primary site with a median follow-up of 14.1 months (5–34 months). Two cases (T1N0M0, T1N2M0) were then treated with local conformal radiation therapy (70 Gy/35 F) with a good tolerance, but the T1N0M0 patient died of local hemorrhage in 22 months after recurrence, and the other one was still alive after 3 months. One patient (T4N1M0) with an intracranial relapse received palliative surgical resection and is still alive. The remaining five cases had only supportive treatment and died of local hemorrhage in an average of 4.4 months (1–7 months) after relapse.

Three patients (T4N1 M0, two T3N2 M0 cases) had nodal recurrence with a median follow-up of 16 months (10–24 months). Two cases underwent surgical resection, among them the T4N1 M0 patient died of second relapse 12 months after the surgical operation, and the other one is currently alive for more than 6 months. The other T3N2 M0 case underwent 4 cycles of salvage chemotherapy and local I^125^ seeds implantation and died of uncontrolled tumor 10 months later.

Thirteen patients (five stage II, five stage III, and three stage IV, resp., all had been N+) had distant metastasis in a median follow-up of 17.9 months (3–38 months). Three cases with multiple liver metastases had no salvage chemotherapy and died of respiratory and circulatory failure in a median of 3.7 months (1–5 months) after relapse. Five patients had bone metastases, among them one case (T2aN1M0) with multiple bone metastases had 6 cycles of chemotherapy with a life prolongation for 31 months but died of tumor progression later and two cases (T3N1M0, T2N2M0) with multiple bone metastases had a rapid disease progression and died 3.5 months later; two other cases had a single spinal metastasis; one (T2bN1M0) of them received surgery and chemotherapy but died of tumor diffusion 35 months after metastasis discovery and the other one (T4N1M0) received local IMRT (60 Gy/30 F) and is still alive for 10 months without disease progression. One patient (T4N1M0) with pathologically confirmed intramedullar metastasis leading to paralysis received local radiation therapy (40 Gy/20 F) and intrathecal chemotherapy and is still alive for 12 months. Four patients had multiorgan metastasis; one case (T1N1M0) died 1 month later, two cases (T1N2M0, T3N1M0) received chemotherapy and died of respiratory and circulatory failure 12 months (7 and 17 months) after metastasis discovery, and the remaining case (T2bN1M0) had just received chemotherapy and radiation therapy for one month and is still alive.

Five patients (three T4, one T3, and one T2b) died of unexplained pharyngeal bleeding in an average follow-up of 8 months (6–12 months). One patient (T4N3aM0) died from a sudden onset of acute brain herniation (an intracranial metastasis could not be excluded) 9 months after radiation therapy. One 76-year-old patient (T1N1M0) had a persistent pharyngeal ulcer and died of exhaustion 12 months after radiation therapy alone.

### 3.4. Survival Analysis

**Figure 1:**
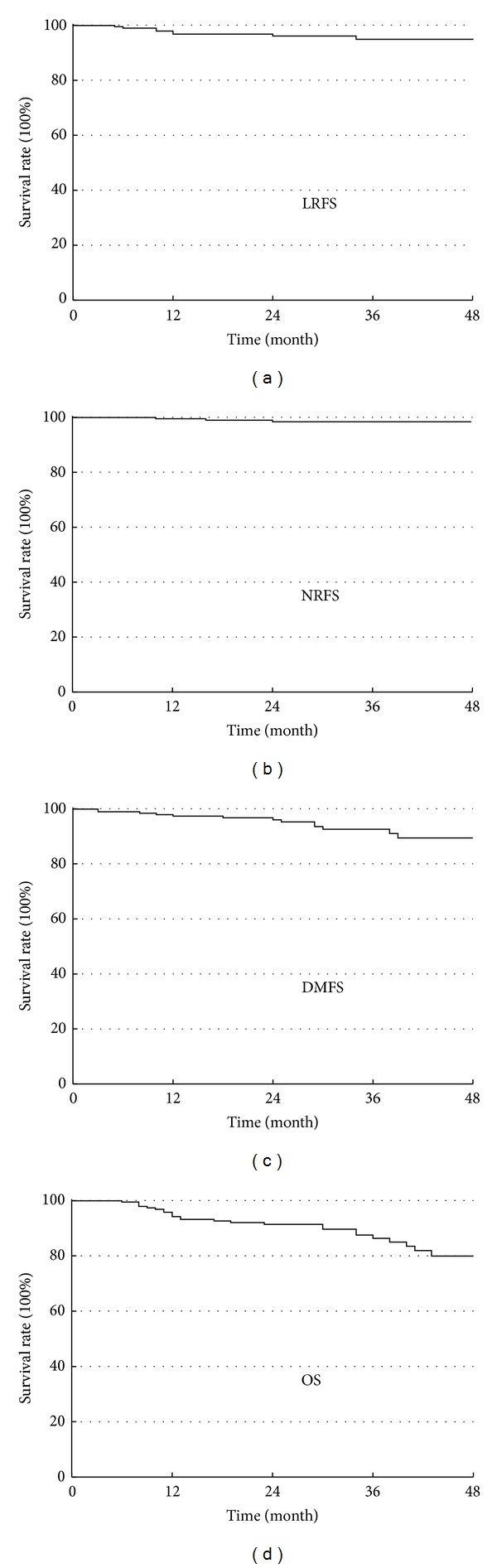
Kaplan-Meier estimate of local relapse-free survival (LRFS), nodal relapse-free survival (NRFS), distant metastases-free survival (DMFS), and overall survival (OS) rates.

For the cohort of this study, the 3-year local relapse-free survival (LRFS), nodal relapse-free survival (NRFS), distant metastases-free survival (DMFS), and overall survival (OS) were 96.1%, 98.2%, 92.0%, and 86.3%, respectively (Figure 1). Three-year follow-up is completed in 74 patients.

Univariate analysis (Table 6) showed that age affected 3-year LRFS (*χ* ^2^ = 4.046, *P* = 0.044), T stage affected 3-year NRFS (*χ* ^2^ = 11.293, *P* = 0.010), age and N stage affected 3-year DMFS (*χ* ^2^ = 6.063, *P* = 0.014; *χ* ^2^ = 8.160, *P* = 0.043), and age and T stage affected 3-year OS (*χ* ^2^ = 6.791, *P* = 0.009; *χ* ^2^ = 10.218, *P* = 0.017). In locally advanced tumors (stages III-IV), cisplatin-based chemotherapy in different periods affected 3-year LRFS and DMFS (*χ* ^2^ = 9.88, *P* = 0.002; *χ* ^2^ = 9.88, *P* = 0.002). Cox multivariate regression analysis showed that age and T stage were independent predictors for 3-year OS (HR = 2.981, 95% CI = 1.201–7.400, *P* = 0.019; HR = 1.620, 95% CI = 1.061–2.474, *P* = 0.026).

## 4. Discussion

IMRT has the ability to deliver high doses of radiation to the target structures while sparing adjacent bystander healthy tissues and has now become the preferred radiation therapy modality. Recent clinical studies showed that IMRT improved local control compared with 2DCRT in NPC [10, 11]. HT is a novel form IMRT with a 6 MV linear accelerator producing a fine beam modulated multileaf collimator. Patients are translated through the bore synchronized with gantry rotation [18]. The technique allows greater conformity of radiation dose to tumor volumes that are in close proximity to critical structures, such as those seen in NPC. In our study the average beam-on time was 456.5 sec, at least 5 min less than that of LINAC-based step-and-shoot IMRT. Our previous dosimetric study of 10 NPC patients found that HT had a better dose homogeneity, steeper gradient, and a reduction for delivered dose to many OARs in comparison with LINAC-based step-and-shoot IMRT [19]. Lee et al. [20] saw similar results when comparing HT plans to those of step-and-shoot IMRT in 20 NPC patients. In addition, they found that the actual treatment time was 8.1 ± 0.6 min (range: 6.7–10.5 min) compared with the simulated treatment time of 13.9 ± 1.3 min (range: 11.2–16.7 min) in step-and-shoot IMRT (*P* < 0.001). Fiorino et al. [21] compared HT plan with that of LINAC-based IMRT using dynamic MLC technique in six patients with locally advanced NPC. They found that the mean dose to the parotids decreased from 30.1 Gy for LINAC-based IMRT to 25.0 Gy for HT. Lu et al. [22] showed that HT plans provided a better conformity index than VMAT and IMRT, with a similar parotid sparing effect compared with VMAT but better than IMRT. However, randomized clinical studies are needed to confirm the superiority of HT. At our center, the whole parotid gland is contoured, and before May 2009 the dose constraint was defined as V30 < 50%. Consequently, the parotid glands received a higher dose (*D*_mean_ = 31.0 Gy for left parotid gland, *D*_mean_ = 30.8 Gy for right parotid gland) in this study. After May 2009 we changed the dose constraint to *D*_mean_ < 28 Gy. In this study, the incidence of submandibular edema was high. The reason may be that HT projects doses from 360 degrees resulting in a larger low-dose subcutaneous region.

Intergroup 0099 established the role for chemoradiotherapy in the treatment of NPC [23]. Concurrent chemoradiotherapy followed by adjuvant chemotherapy is still the standard of care for locoregionally advanced NPC. The role of induction chemotherapy in addition to concurrent chemotherapy remains to be defined and is currently not the standard of care. Four meta-analyses investigating the incorporation of chemotherapy with radiation therapy in NPC have been published. All demonstrated that chemotherapy was beneficial over radiation therapy alone, with the primary benefit seen with concurrent chemotherapy scheduling [24–27]. The Meta-Analysis of Chemotherapy in Nasopharynx Carcinoma (MAC-NPC) meta-analysis performed through the Cochrane Review system was the only meta-analysis for which individual patient data from eight randomized trials was analyzed. This study found that chemotherapy provided a 6% absolute survival benefit at 5 years and an event-free survival benefit of 10% at 5 years. Importantly, timing was highly significant with OS (*P* = 0.005), with concurrent chemoradiotherapy providing the majority of the benefit in survival [26]. In our cohort, univariate analysis confirmed that cisplatin-based chemotherapy in different periods improved 3-year LRFS and DMFS in stage III-IV patients (*P* = 0.002 and *P* = 0.002, resp.). However, chemotherapy—especially concurrent chemotherapy—increases the incidence of treatment-related side effects resulting in a poor treatment compliance of only 60%~70%. In the study of Lee et al. [28], despite a statistically significant reduction in deaths because of cancer progression, the gain in the OS was not statistically significant because deaths due to toxicity or incidental causes increased by concurrent-adjuvant chemotherapy in locally advanced NPC patients. In our study, acute radiation related side effects were mainly grade 1 or 2. Grade 3 and greater toxicities were rarely noted (Table 4). In our cohort, eighty-five patients were treated with concurrent chemoradiotherapy (with or without anti-EGFR Mab treatment); only one case did not finish chemotherapy and two other cases needed a reduction of drug dose while the other patients (96.5%) fully finished the treatment protocol. This can be explained by a strict patient screening and lower drug doses. Kodaira et al. [13] also studied the feasibility of HT in 20 NPC patients among whom 90% received a concurrent chemotherapy; the incident of grade 3 or more leucopenia, skin reaction, and stomatitis (45%, 40% and 55%, resp.) were higher than that noted in our study. In comparison with the outcomes of IMRT for NPC in different centers listed in Table 7, our outcome is comparable with the 3-year locoregional relapse-free survival or DMFS of more than 90% and OS more than 80%, but a long-time follow-up is needed to confirm the long-term outcome. Univariate analysis showed that age affected 3-year LRFS, T stage affected 3-year NRFS, age and N stage affected 3-year DMFS, and age and T stage affected 3-year OS. Cox multivariate regression analysis showed that age and T stage were independent predictors for 3-year OS.

In recent years, the successful application of molecular-targeted drugs, particularly anti-EGFR Mab (including cetuximab and nimotuzumab), has allowed a new choice in the concurrent therapy in head and neck cancer treatment [30–33]. Bonner et al. [30] showed significant survival benefits for nonnasopharyngeal head and neck cancer when cetuximab was added to radiation therapy in a phase III trial. Chan et al. [31] reported the outcome of a multicenter phase II study where cetuximab in combination with carboplatin was administrated to 60 recurrent or metastatic NPC patients after failure with initial cisplatin-based chemotherapy, with 7 PR (11.7%) and 29 SD (48.3%). Huang et al. [32] reported that nimotuzumab in combination with radiation therapy was effective and well tolerated for locally advanced NPC in a multicenter phase II trial. In our cohort, fifty-five patients (28.9%) had an anti-EGFR Mab treatment as the only concurrent therapy and noted an incidence of acute side effects comparable with the patients receiving radiation therapy alone, but no benefit was detected by statistical analysis for stage III-IV patients, so the doubt concerning the effect of anti-EGFR Mab in comparison with chemotherapy, especially when it is added to concurrent chemoradiotherapy, needs to be clarified by long-term clinical observation.

Through the analysis of failure cases in our cohort, we could see that a high percentage of failures were local recurrence (8 of 31 total failures). There may be two main reasons. First, the radiation dose may have been insufficient; five T2b-T4 cases with relatively large GTV volumes had an in-field recurrence (two cases with anti-EGFR Mab, one case with anti-EGFR Mab and adjuvant chemotherapy, and two cases with concurrent and adjuvant chemotherapy), suggesting the need for a higher radiation dose. Since September 2011, we have increased the pGTVnx dose from 70 Gy in 33 fractions to 67.5 Gy in 30 fractions, hoping to improve the local control by raising fractional dose. Second, the GTVnx-to-pGTVnx margin may have been too narrow; except in-field failure, three cases developed marginal recurrence. Ng et al. [8] analyzed the failure patterns in 193 stage III-IV NPC patients treated with IMRT and found that 3 of 16 local failures were marginal. We believed that this was most likely due to the small GTVnx-to-pGTVnx expansion margin and the setup error which was not corrected by daily MVCT image-guidance. Therefore, since March 2009, the GTVnx-to-pGTVnx margin was increased from 3 mm to 5 mm and daily pretreatment image-guidance was performed in our center. Thereafter, the marginal failure declined from 3.8% (two among fifty-three cases) to 1.1% (one among ninety-five cases). However, statistical analysis failed to detect a significant difference between the two treatment strategies for 3-year LRFS, probably because of the low overall probability of the marginal failure.

In NPC, appropriate salvage therapy can achieve radical cure or prolong survival after locoregional failure or distant metastasis. Zhou et al. [34] reirradiated 53 locally recurrent patients with IMRT (67.9 Gy) combined with cisplatin-based chemotherapy. Their 2-year OS and progression-free survival were 58.7% and 52.3%, respectively; but reirradiation caused severe stomatitis and bleeding and 10 cases (45.5%) died of bleeding among all 22 deaths. Goto et al. [35] using HT with concurrent chemotherapy reirradiated 50 locally recurrent patients and got similar results. In addition, salvage surgery and chemotherapy are also effective treatments. Chang et al. [36] reported 38 primary recurrent NPC patients who underwent salvage surgery with curative intention via the facial translocation approach, with the 3-year OS and LCR of 60% and 72.8%, respectively. Chan et al. [31] conducted a phase II study using C225 in combination with carboplatin in patients with recurrent or metastatic NPC, with the PR and SD of 11.7% and 48.3%, respectively. In our cohort, patients with locoregional recurrence or metastasis receiving salvage therapy survived 16 months longer than those without salvage therapy (*P* = 0.006), showing the significance of salvage treatment in recurrent or metastatic NPC.

In our cohort, lethal pharyngeal hemorrhage took a relatively high proportion among all failures (five of thirty-one). Nasal cavity and nasopharyngeal bleeding with little volume, which is easy to control, is a common complication of radiation therapy. However, all our five cases had T3-4 stage tumors with carotid artery invasion. Due to acute onset, these patients lost rescue time and local tumor recurrence could not be excluded. Since January 2011, we have advised the patients with bleeding risk to refrain from strongly blowing their noses; in no case has a patient died of hemorrhage since.

## 5. Conclusions

Helical tomotherapy for NPC patients achieved excellent 3-year locoregional control, distant metastasis-free survival, and overall survival, with relatively minor acute and late toxicities. Age and T stage were the main prognostic factors. Preventive measures against pharyngeal bleeding are needed in patients with carotid artery invasive tumor.

## Figures and Tables

**Table 1:** Distributions of patients according to the UICC 2002 staging system.

Stage	N_0_	N_1_	N_2_	N_3_	Total
T_1_	16	27	15	3	61
T_2_	13	24	22	2	61
T_3_	8	11	18	3	40
T_4_	3	10	11	4	28

Total	40	72	66	12	190

**Table 2:** Patient's characteristics.

Characteristics	Patients	
	Number	%
Age (median)	10–81 (44)	
Male	144	75.8
Female	46	24.2
ECOG performance status		
0	57	30.0
1	113	59.5
2	20	10.5
Pathology		
WHO type I	3	2.5
WHO type II/III	118	97.5
UICC 2002 stage		
I	16	8.4
IIa-b	64	33.7
III	71	37.4
IVa-b	39	20.5

**Table 3:** Chemotherapy and anti-EGFR monoclonal antibody (Mab) treatment.

Chemotherapy	Concurrent anti-EGFR Mab treatment		Total
	+	−	
None	30	31	61
NACT∗	1	5	6
CCT	22	26	48
ACT	21	14	35
NACT + CCT	0	10	10
CCT + ACT	2	24	26
NACT + CCT + ACT	1	0	1
NACT + ACT	3	0	3

Total	80	110	190

*NACT: neoadjuvant chemotherapy, CCT: concurrent chemotherapy, and ACT: adjuvant chemotherapy.

**Table 4:** Acute radiation related toxicities.

Acute toxicities	Grade 0 (%)	Grade 1 (%)	Grade 2 (%)	Grade 3 (%)	Grade 4 (%)
Skin reaction	7 (3.7)	137 (72.1)	37 (19.5)	9 (4.7)	0 (0)
Mucositis	4 (2.1)	72 (37.9)	108 (56.8)	6 (3.2)	0 (0)
Xerostomia	9 (4.74)	100 (52.63)	81 (42.63)	0 (0)	0 (0)
Esophagitis-tracheitis	7 (3.7)	83 (43.7)	99 (52.1)	1 (0.5)	0 (0)
Leucopenia	86 (45.3)	42 (22.1)	50 (26.3)	10 (5.3)	2 (1.0)
Anemia	175 (92.1)	14 (7.4)	1 (0.5)	0 (0)	0 (0)
Thrombocytopenia	180 (94.7)	7 (3.7)	2 (1.1)	1 (0.5)	0 (0)

**Table 5:** Distributions of failure cases.

Patterns of failure	Numbers of patients		Total (*n*, %)
	Alive	Died	
Local recurrence	2	6	8 (25.8)
Nodal recurrence	1	2	3 (9.7)
Distant metastasis	3	10	13 (41.9)
Pharyngeal bleeding	0	5	5 (16.1)
Others	0	2	2 (6.5)

Total (*n*, %)	6 (19.4)	25 (80.6)	31 (100)

**Table 6:** Log-rank test for univariate analysis.

Factor	3-year LRFS			3-year NRFS			3-year DMFS			3-year OS		
Age (y)												
≤50	94.8%	*χ* ^2^ = 4.046	**P** = 0.044	94.8%	*χ* ^2^ = 1.211	*P* = 0.271	91.5%	*χ* ^2^ = 6.063	**P** = 0.014	83.1%	*χ* ^2^ = 6.791	**P** = 0.009
>50	80.8%			100.0%			70.4%			58.8%		
Gender												
Male	93.7%	*χ* ^2^ = 2.848	*P* = 0.091	96.8%	*χ* ^2^ = 0.202	*P* = 0.653	82.1%	*χ* ^2^ = 1.815	*P* = 0.178	74.0%	*χ* ^2^ = 0.135	*P* = 0.713
Female	81.0%			94.4%			94.7%			77.3%		
T Stage												
T1	88.9%	*χ* ^2^ = 7.424	*P* = 0.060	100.0%	*χ* ^2^ = 11.293	**P** = 0.010	89.5%	*χ* ^2^ = 1.357	*P* = 0.716	81.0%	*χ* ^2^ = 10.218	**P** = 0.017
T2	97.7%			100.0%			87.0%			85.1%		
T3	80.0%			80.0%			77.8%			53.8%		
T4	70.5%			90.9%			75.0%			55.6%		
N Stage												
N0	85.7%	*χ* ^2^ = 1287	*P* = 0.732	100.0%	*χ* ^2^ = 6.116	*P* = 0.160	100.0%	*χ* ^2^ = 8.160	**P** = 0.043	85.7%	*χ* ^2^ = 1.765	*P* = 0.622
N1	90.0%			100.0%			72.7%			70.3%		
N2	92.6%			92.9%			86.2%			73.5%		
N3	100.0%			83.3%			100.0%			71.4%		
Node category												
N+	85.7%	*χ* ^2^ = 0.722	*P* = 0.395	100.0%	*χ* ^2^ = 0.871	*P* = 0.351	100.0%	*χ* ^2^ = 3.782	*P* = 0.052	85.7%	*χ* ^2^ = 1.700	*P* = 0.192
N−	92.1%			95.2%			80.9%			71.8%		
UICC Stage												
I	83.3%	*χ* ^2^ = 6.097	*P* = 0.107	100.0%	*χ* ^2^ = 3.235	*P* = 0.357	100.0%	*χ* ^2^ = 1.634	*P* = 0.652	82.5%	*χ* ^2^ = 3.645	*P* = 0.302
II	100.0%			100.0%			82.1%			85.0%		
III	86.7%			93.1%			83.3%			96.4%		
IV	80.0%			90.9%			91.7%			66.7%		
IGRT and GTVnx to pGTVnx margin												
Every day and 5 mm	92.5%	*χ* ^2^ = 1.098	*P* = 0.295	100%	*χ* ^2^ = 1.096	*P* = 0.295	86.8%	*χ* ^2^ = 1.409	*P* = 0.306	81.1%	*χ* ^2^ = 0.299	*P* = 0.585
Every week and 3 mm	96.8%			97.9%			93.7%			86.3%		
Chemotherapy was performed or not in stage III-IV patients												
No	57.1%	*χ* ^2^ = 4.563	**P** = 0.033	100.0%	*χ* ^2^ = 0.406	*P* = 0.524	57.1%	*χ* ^2^ = 4.563	**P** = 0.033	55.6%	*χ* ^2^ = 0.648	*P* = 0.421
Yes	90.0%			92.1%			90.0%			71.7%		
Concurrent chemotherapy was performed or not in stage III-IV patients												
No	81.5%	*χ* ^2^ = 0.577	*P* = 0.447	91.7%	*χ* ^2^ = 0.150	*P* = 0.699	81.5%	*χ* ^2^ = 0.577	*P* = 0.447	70.0%	*χ* ^2^ = 0.047	*P* = 0.829
Yes	90.0%			94.7%			90.0%			68.0%		
Anti-EGFR monoclonal antibody was used or not in stage III-IV patients												
No	84.0%	*χ* ^2^ = 0.071	*P* = 0.789	91.3%	*χ* ^2^ = 0.219	*P* = 0.640	84.0%	*χ* ^2^ = 0.071	*P* = 0.789	65.6%	*χ* ^2^ = 0.399	*P* = 0.528
Yes	86.4%			95.0%			86.4%			73.9%		

**Table 7:** Outcomes of IMRT for NPC in published series.

Author (year)	Number of patients	Treatment period	Median follow-up (m)	Stage	Treatment arms	Time point (y)	LC (LRFS)	RC (NRFS)	DMFS	OS
Tham et al. (2009) [6]	195	2002–2005	37	I–IVB	RT or CRT	3	93%	NR	89%	94%
Lee et al. (2009) [5]	68	2003–2005	30	I–IVB	RT or CRT	2	93%	91%	85%	80%
Su et al. (2012) [9]	198	2001–2008	51	I–IIB	RT	5	98%	NR	98%	97%∗
Lai et al. (2011) [10]	512	2003–2006	53	I–IVB	RT or CRT	5	93%	97%	84%	NR
Lin et al. (2010) [7]	370	2003–2007	31	IIB–IVB	RT or CRT	3	95%	97%	81%	89%
Ng et al. (2011) [8]	193	2005–2007	30	I–IVB	RT or CRT	2	95%	96%	90%	92%
Peng et al. (2012) [11]	306	2003–2008	42	I–IVB	RT or CRT	5	90.5%	91.7%	NR	79.6%
Wang et al. (2012) [29]	138	2006–2009	23	I–IVB	RT or CRT	3	94%	96%	80%	83%
This study	190	2007–2012	32	I–IVB	RT or CRT or ART	3	96%	98%	92%	86%

RT: radiation therapy alone; CRT: chemoradiotherapy; ART: radiation therapy concurrent with anti-EGFR Mab therapy; LC: local control; LRFS: local relapse-free survival; RC: regional control; NRFS: nodal relapse-free survival; DMFS: distant metastases-free survival; OS: overall survival; NR: not reported; ∗disease-specific survival.

## Abbreviations

NPC: Nasopharyngeal carcinoma
IMRT: Intensity modulated radiation therapy
HT: Helical tomotherapy
PLA: People's Liberation Army
CT: Computed tomography
MRI: Magnetic resonance imaging
PET: Positron emission tomography
GTVnx: Gross target volume of the primary tumor
GTVnd: Gross target volume of metastatic lymph nodes
CTV: Clinical target volume
PTV: Planning target volume
OAR: Organs at risk
RTOG: Radiation therapy oncology group
MVCT: Megavoltage computed tomography
EGFR: Epithelial growth factor receptor
DDP: Cisplatin
Mab: Monoclonal antibody
EORTC: European Organization for Research and Treatment of Cancer
DXT: Docetaxel
*D*_mean_: Mean doses
LRFS: Local relapse-free survival
NRFS: Nodal relapse-free survival
DMFS: Distant metastases-free survival
OS: Overall survival
HR: Hazard ratio
CI: Confidence interval
2DCRT: Two-dimensional conventional radiation therapy
LINACL: Linear accelerator
MLC: Multileaf collimator
VMAT: Volumetric modulated arc therapy
PR: Partial response
CR: Complete response
SD: Stable disease
PD: Progressive disease
LCR: Local control rate.
